# Medications and medical costs for diabetes patients with or without chronic respiratory disease in Beijing, China: A retrospective study

**DOI:** 10.3389/fendo.2022.980982

**Published:** 2022-08-26

**Authors:** Jingtao Qiao, Zheng Tan, Xiaomao Xu, Yan Zhou, Weihao Wang, Jingyi Luo, Jingwen Fan, Qi Pan, Lixin Guo

**Affiliations:** ^1^ Department of Endocrinology, Beijing Hospital, National Center of Gerontology, Institute of Geriatric Medicine, Chinese Academy of Medical Sciences, Beijing, China; ^2^ Department of Respiratory and Critical Care Medicine, Beijing Hospital, National Center of Gerontology, Institute of Geriatric Medicine, Chinese Academy of Medical Sciences, Beijing, China

**Keywords:** chronic respiratory disease (CRD), diabetes mellitus, hypoglycemic therapy, medical cost, medications

## Abstract

**Aims:**

The cost of drug regimens prescribed to Chinese patients has not been evaluated. This study aims to evaluate the medical costs and hypoglycemic agents for diabetes mellitus patients with or without chronic respiratory disease in Beijing, and to investigate the changes in the costs and number of antidiabetic medications used for diabetes patients with chronic respiratory disease from 2016 to 2018.

**Methods:**

This observational, retrospective study included diabetes patients with outpatient medication records from Beijing Medical Insurance between 2016 and 2018. The medications, including hypoglycemic and nonhypoglycemic drugs, insulin dosage, comorbidities, diabetes-related complications, treatment strategies, and annual medical costs, were recorded.

**Results:**

This study included 2,853,036 diabetes patients from 2016 to 2018. About 18.95%–20.53% of patients with chronic respiratory disease were predominantly distributed among those aged 45–84 years (88.7%–89.1%). Diabetes patients with chronic respiratory disease used more medications (4.48 ± 2.41 vs. 3.76 ± 2.33) and had higher total annual drug costs (¥12,286 ± 10,385 vs. ¥9700 ± 9202) to treat more comorbidities (2.52 ± 1.53 vs. 2.05 ± 1.85) than those without chronic respiratory disease (*p* <.0001, respectively). From 2016 to 2018, diabetes patients with chronic respiratory disease had a 4.2% increase in medication, a 1.9% decrease in comorbidities, and a 5.4% decrease in total annual drug costs.

**Conclusions:**

In summary, diabetes patients with chronic respiratory disease had more comorbidities, required more hypoglycemic drugs, and had higher medical costs. During 2016–2018, diabetes patients with chronic respiratory disease used more medications and spent less money on medical care.

## Introduction

Diabetes mellitus (DM) is a chronic, noncommunicable disease that causes premature death and a poor quality of life. Diabetes-related comorbidities and complications, such as hypoglycemia, diabetic kidney disease (DKD), diabetic retinopathy (DR), diabetic peripheral neuropathy (DPN), and others, impose a growing economic burden on patients, families, and the national health care system worldwide (1–3), resulting in two to three times higher medical costs than people without diabetes (4, 5). The International Diabetes Federation (IDF) estimated 537 million diabetes patients globally in 2021, causing nearly $1 trillion in expense, or 9% of all health care expenditure (6).

Over the past several decades, a growing amount of research has focused on the efficacy and cost-effectiveness of diabetes treatment strategies in different countries and areas (1, 7–9). Generally, the direct medical cost (DMC) is generally influenced by numbers of diabetes-related complications (1, 3) and is also associated with the length of hospital stay (8). In China, IDF data showed that 140 million people had diabetes in 2021, which is expected to rise to 174.4 million by 2045 (6). About 49.2% of patients treated got adequate control of their blood glucose levels (10). However, management of blood glucose levels is even worse in rural areas in China (11), which means the medical burden is higher than in published data.

A previous study uncovered that diabetes patients used more medical costs when suffering end-stage renal disease, congestive heart failure, and myocardial infarction (12). However, in recent years, according to recent studies, diabetes patients are at a higher risk of chronic respiratory disease (CRD) than nondiabetes patients. Mannino et al. report that chronic obstructive pulmonary disease (COPD) is associated with an increased risk of diabetes and higher hospitalization and mortality rates (13). A related study conducted in an Asian series showed that there was a higher risk of death in diabetes patients with CRD significantly (odds ratio [OR] = 2.03) (14). Previous research uncovered that insulin resistance or diabetes may contribute to a decline in lung function (15, 16). Wang et al. uncovered the significant association between DM patients and the risk of total CRD mortality, in particular, from respiratory infection (17) as well as in tuberculosis (TB) (18), and sleep apnea–hypopnea syndrome (SAHS) (19). A higher risk of pneumococcal disease was observed in DM patients as well (20). Insulin resistance or diabetes may contribute to a decline in lung function (21).

Meanwhile, the average medical expenditure for pneumonia was significantly higher in diabetes patients than in nondiabetes patients (22). A previous study investigated the economic benefit of influenza vaccination in diabetes patients. CRD, an overlooked comorbidity of diabetes, is a major factor in diabetes patients’ antidiabetic medications and medical expenditure. However, the medical expenses and antidiabetic agents for diabetes patients with CRD remain unanswered.

In this study, we conduct a cost-effectiveness analysis to analyze the difference in hypoglycemic strategies and medical costs between diabetes with CRD and diabetes without CRD and to investigate the changes of antidiabetic medications and costs in diabetes with CRD, using medical insurance data from 2016 to 2018 in Beijing, China.

## Patients and methods

### Study design and settings

We performed a retrospective, observational study to analyze the difference in antidiabetic medications and costs between diabetes patients with or without the CRD series. This study was approved by the ethics committee of Beijing Hospital.

### Study patients and data collection

This study enrolled diabetes patients with outpatient medical records from Beijing Medical Insurance between 2016 and 2018. All patients were over the age of 16. Diabetes diagnoses were confirmed *via* the World Health Organization (WHO) 1999 criteria. Patients without a continuous record of a prescription for more than 2 months would be excluded under the current medical insurance system, in which doctors in Beijing prescribe hypoglycemic prescriptions for fewer than 30 days in Beijing, and patients must return to the clinic to take medications.

We collected the information from the Beijing Medical Insurance database, including ICD diagnosis, age, gender, course, medications (hypoglycemic and nonhypoglycemic drugs), insulin dosage, and medical costs.

### Definitions of comorbidities and complications

The CRD includes asthma, COPD, tracheitis/bronchitis, TB, and lung cancer. Considering the indiscriminate use of antibiotics, acute pulmonary infection was excluded from this study.

Comorbidities of diabetes patients include hypertension, coronary atherosclerotic heart disease (CAD), dyslipidemia, stroke, chronic lung disease, and osteoporosis. Diabetes-related complications were as follows: DPN, DKD, DR, and diabetic angiopathies (DAs).

### Hypoglycemic treatments and stratification

Of the medications containing hypoglycemic and nonhypoglycemic drugs, hypoglycemic drugs included insulin and oral antidiabetic drugs (OADs) that contain α-glucosidase inhibitors (AGIs), metformin, sulfonylurea (SUs), thiazolidinediones, and glinides. Insulin included fast-, short-, intermediate-, and long-acting and premixed dosages. The diabetes treatment strategy classes included the following: 1) Monotherapy: patients who received only one recorded hypoglycemic drug prescription in the past 1 year; 2) oral combination therapy: patients who received two or more OADs from different classes in the past 1 year in different classes; and 3) combination of oral and insulin therapy: patients who received at least one insulin and at least one OAD medications in the past 1 year.

### Statistical analysis

Statistical analysis was performed with SAS software, version 9.4 (SAS Institute, Inc). Data with a normal distribution were expressed as the mean ± standard deviation (x ± SD) and were statistically compared using the *Wilcoxon rank sum test*, such as the percentage distributions of the number of medications, comorbidities, drug costs, and diseases diagnosed, such as the prevalence of hypertension, dyslipidemia, stroke, *etc.* When the distributions of variables were overdispersed, we used the *negative binomial model* and the *log link function*. We used the *multivariable regression model* to control for confounding factors. The categorical variables data, which were calculated as a frequency, were presented as numbers and percentages, such as the insulin usage rate, the gender/age ratio and *the chi-square test* (*χ* ^2^), and *Fisher’s exact test* was used for comparison; *p* <.05 was considered statistically significant.

## Results

### Baseline characteristics

**Figure 1:**
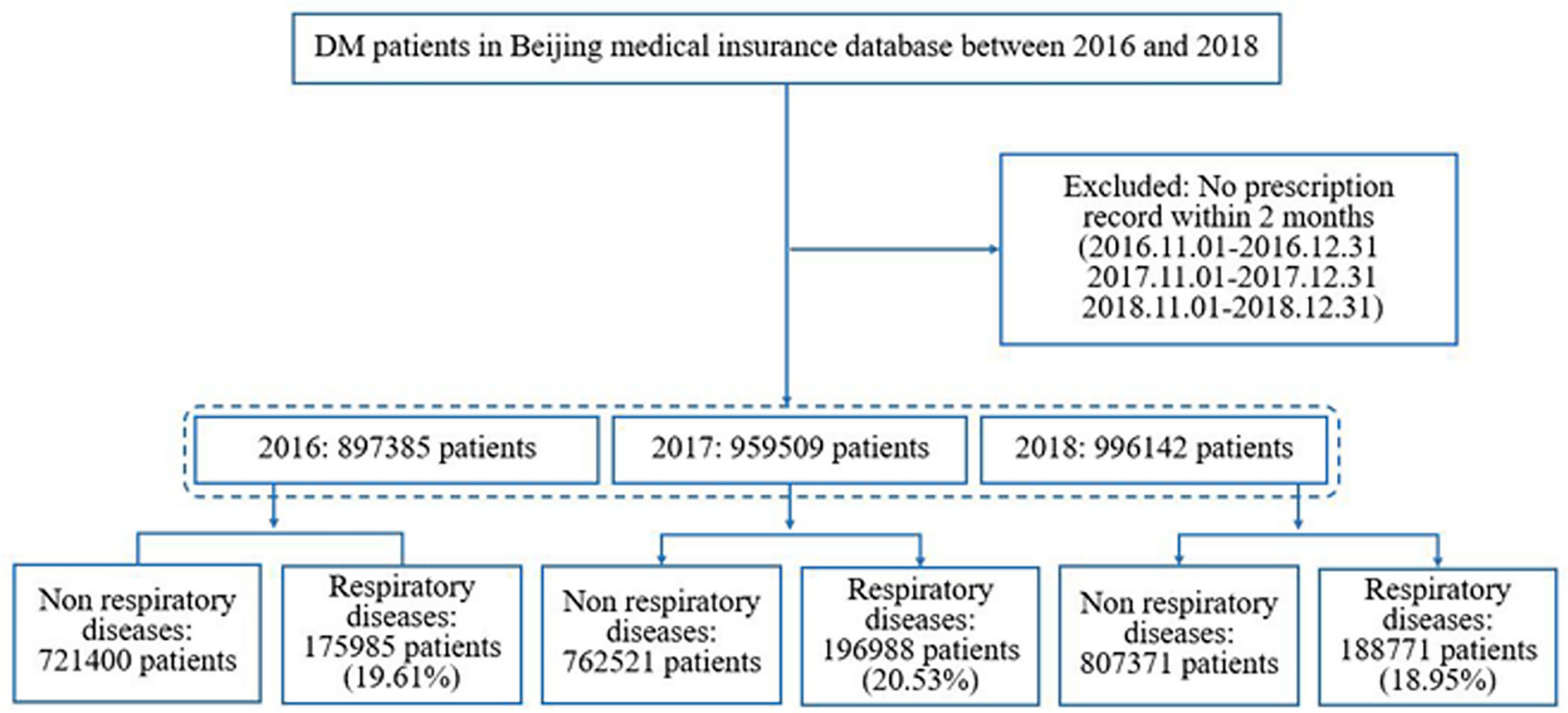
Patient enrollment flow chart.

This study included 2,853,036 diabetes patients (897,385 diabetes patients in 2016; 959,509 diabetes patients in 2017; and 996,142 diabetes patients in 2018). **Figure 1** shows a flow chart of patient enrollment. Among them, 18.95%–20.53% of diabetes patients had CRD (175,985/897,385 (19.61%) in 2016; 196,988/959,509 (20.53%) in 2017; and 188,771/996,142 (18.95%) in 2018). The proportion of diabetes patients with CRD varied significantly by age group with most patients aged 45–84 years (88.7%–89.1%) affected. **Table 1** shows the detailed proportions of the different age groups in the two series. Compared with non-CRD patients, the category with comorbid CRD had more female patients (52.3% vs. 49.1% in 2016, 51.6% vs. 48.1% in 2017, and 50.3% vs. 47.0% in 2018), whereas men outnumbered women in all diabetes populations (50.3% vs. 49.7% in 2016, 51.2% vs. 48.8% in 2017, and 52.4% vs 47.6% in 2018). They also had a higher prevalence of hypertension (69.5% vs. 59.4%), coronary heart disease (59.0% vs. 46.4%), dyslipidemia (51% vs. 46.9%), stroke (25.2% vs. 18.5%), osteoporosis (17.6% vs. 12.4%), DPN (16.5% vs. 13.0%), DKD (4.8% vs. 3.4%), DR (4.7% vs. 4.1%), and DA (3.7% vs. 2.9%) with significant differences, respectively (all *p’*s <.0001 from 2016 to 2018; we only list data in 2018 in the text) (**Table 1**).

**Table 1:** Demographic characteristic in diabetes patients in Beijing between 2016 and 2018.

			Non-CRD			CRD		χ2 test, 2016	χ2 test, 2017	χ2 test, 2018
		2016	2017	2018	2016	2017	2018	*p value*	*p value*	*p value*
Age group (%)								<0.0001	<0.0001	<0.0001
	16-44y	63185 (8.8)	67359 (8.8)	69643 (8.6)	10958 (6.2)	12086 (6.1)	10964 (5.8)			
	45-64y	387852 (53.8)	401875 (52.7)	414940 (51.4)	90796 (51.6)	100374 (51.0)	94823 (50.2)			
	65-84y	255018 (35.4)	275037 (36.1)	300605 (37.2)	69145 (39.3)	78136 (39.7)	76176 (40.4)			
	≥85y	15345 (2.1)	18250 (2.4)	22183 (2.7)	5086 (2.9)	6392 (3.2)	6808 (3.6)			
Gender (%)								<0.0001	<0.0001	<0.0001
	Male	367397 (50.9)	395503 (51.9)	428269 (53.0)	84029 (47.7)	95388 (48.4)	93792 (49.7)			
	Female	354003 (49.1)	367018 (48.1)	379102 (47.0)	91956 (52.3)	101600 (51.6)	94979 (50.3)			
Hypertension (%)		427726 (59.3)	456660 (59.9)	479686 (59.4)	122872 (69.8)	138251 (70.2)	131241 (69.5)	<0.0001	<0.0001	<0.0001
CAD (%)		337014 (46.7)	363725 (47.7)	374943 (46.4)	104423 (59.3)	119080 (60.5)	111379 (59.0)	<0.0001	<0.0001	<0.0001
Dyslipidemia (%)		318894 (44.2)	351301 (46.1)	378952 (46.9)	84176 (47.8)	99228 (50.4)	96352 (51.0)	<0.0001	<0.0001	<0.0001
Stroke (%)		149883 (20.8)	152698 (20.0)	149058 (18.5)	50260 (28.6)	54710 (27.8)	47653 (25.2)	<0.0001	<0.0001	<0.0001
Osteoporosis (%)		97680 (13.5)	102452 (13.4)	99753 (12.4)	34888 (19.8)	38222 (19.4)	33226 (17.6)	<0.0001	<0.0001	<0.0001
DPN (%)		87774 (12.2)	92879 (12.2)	105051 (13.0)	27725 (15.8)	30682 (15.6)	31105 (16.5)	<0.0001	<0.0001	<0.0001
DKD (%)		32223 (4.5)	29889 (3.9)	27819 (3.4)	10856 (6.2)	10890 (5.5)	9072 (4.8)	<0.0001	<0.0001	<0.0001
DR (%)		32462 (4.5)	33228 (4.4)	32881 (4.1)	9435 (5.4)	10161 (5.2)	8860 (4.7)	<0.0001	<0.0001	<0.0001
DA (%)		22320 (3.1)	22294 (2.9)	23248 (2.9)	6925 (3.9)	7566 (3.8)	7006 (3.7)	<0.0001	<0.0001	<0.0001

CRD, chronic respiratory disease; CAD, coronary atherosclerotic heart disease; DPN, diabetic peripheral neuropathy; DKD, diabetic kidney disease; DR, diabetic retinopathy; DA, diabetic angiopathies.

### Differences in medications and costs between diabetes patients with CRD and without CRD

When we focused on the medications and costs of diabetes patients, we discovered that diabetes patients with respiratory diseases used more medications and had higher total annual drug costs than non-CRD patients, including hypoglycemic drugs (1.93 ± 1.08 vs. 1.71 ± 1.08 medications and ¥5906 ± 7111 vs. ¥5188 ± 6985, *p* <.0001, respectively) and nonhypoglycemic drugs (2.55 ± 1.93 vs. 2.05 ± 1.85 medications and ¥6380 ± 6670 vs. ¥ 4512 ± 5288, *p* <.0001, respectively). Diabetes patients with CRD had 2.52 ± 1.53 comorbidities (0.30 ± 0.59 related to hyperglycemia and 2.22 ± 1.32 not related to hyperglycemia), more than the non-CRD group, which had 2.07 ± 1.87 comorbidities (0.23 ± 0.53 as a consequence of hyperglycemia and 1.84 ± 1.33 not related to hyperglycemia, *p* <.0001). As expected, the total annual drug cost/number of drugs had a statistical difference with the cost in the diabetes series with CRD being higher (¥2706 ± 2106 vs ¥2493 ± 2518, *p* <.0001), not only in the total annual hypoglycemic drug cost/number of drugs but also in nonhypoglycemic drugs (¥2800 ± 3058 vs. ¥2624 ± 3228, ¥2148 ± 2104 vs. ¥1661 ± 1837 *p* <.0001, respectively) (**Table 2**).

**Table 2:** The difference in medications, comorbidities, and drug costs between non-CRD and CRD in diabetes.

		Non-CRD			CRD		2016	2017	2018
	2016	2017	2018	2016	2017	2018	*p* value	*p* value	*p* value
Number of medications	3.59 ± 2.33	3.71 ± 2.35	3.76 ± 2.33	4.30 ± 2.40	4.44 ± 2.45	4.48 ± 2.41	<0.0001	<0.0001	<0.0001
Hypoglycemic drugs	1.57 ± 1.03	1.65 ± 1.06	1.71 ± 1.08	1.79 ± 1.05	1.87 ± 1.07	1.93 ± 1.08	<0.0001	<0.0001	<0.0001
Nonhypoglycemic drugs	2.02 ± 1.87	2.07 ± 1.87	2.05 ± 1.85	2.51 ± 1.94	2.57 ± 1.96	2.55 ± 1.93	<0.0001	<0.0001	<0.0001
Number of comorbidities	2.09 ± 1.54	2.11 ± 1.53	2.07 ± 1.50	2.57 ± 1.58	2.58 ± 1.57	2.52 ± 1.53	<0.0001	<0.0001	<0.0001
Glycemic diseases	0.24 ± 0.54	0.23 ± 0.53	0.23 ± 0.53	0.31 ± 0.61	0.30 ± 0.60	0.30 ± 0.59	<0.0001	<0.0001	<0.0001
Nonglycemic diseases	1.85 ± 1.36	1.87 ± 1.36	1.84 ± 1.33	2.25 ± 1.36	2.28 ± 1.35	2.22 ± 1.32	<0.0001	<0.0001	<0.0001
Total annual drug cost, ¥	10,561 ± 10,748	9,530 ± 9,398	9,700 ± 9,202	12,989 ± 11,673	12,160 ± 10,870	12,286 ± 10,385	<0.0001	<0.0001	<0.0001
Hypoglycemic drugs	5,398 ± 7,993	5,002 ± 7,033	5,188 ± 6,985	6,116 ± 7,860	5,763 ± 7,327	5,906 ± 7,111	<0.0001	<0.0001	<0.0001
Nonhypoglycemic drugs	5,163 ± 6,345	4,528 ± 5,452	4,512 ± 5,288	6,873 ± 7,582	6,397 ± 7,028	6,380 ± 6,670	<0.0001	<0.0001	<0.0001
Total annual cost/drug, ¥	2,814 ± 3,053	2,461 ± 2,447	2,493 ± 2,518	2,955 ± 2,530	2,682 ± 2,199	2,706 ± 2,106	<0.0001	<0.0001	<0.0001
Cost/hypoglycemic drug/)	2,903 ± 3,901	2,591 ± 3,242	2,624 ± 3,228	3,063 ± 3,594	2,795 ± 3,305	2,800 ± 3,058	<0.0001	<0.0001	<0.0001
Cost/nonhypoglycemic drug	1,891 ± 2,253	1,644 ± 1,885	1,661 ± 1,837	2,297 ± 2,350	2,100 ± 2,132	2,148 ± 2,104	<0.0001	<0.0001	<0.0001

CRD, chronic respiratory disease.

Diabetes patients with CRD took more medications than non-CRD patients regardless of age, gender, or whether they had dyslipidemia/CAD/osteoporosis/DPN/DKD/DR/DA (**Table 3**). Correspondingly, the diabetes patients with respiratory disease paid more in total annual drug costs (**Table 4**). In general, diabetes patients with CRD need more medications and drug costs for treatment regardless of how many complications they have. The drug cost differential between the two groups decreases as the number of complications or comorbidities increases—the respiratory disease group with four complications spent ¥20,307 ± 16,277, whereas non-CRD patients spent ¥18,715 ± 16,682.

**Table 3:** Numbers of medications in Stratified Patient Groups.

				Non-CRD				CRD		*Wilcoxon test*
		N	Mean	Adjusted Mean^a^	SD	N	Mean	Adjusted Mean^a^	SD	*p* value
Age										
	≥85y	55,778	3.79	3.80	2.31	18,286	4.37	4.36	2.41	<0.0001
	16-44y	200,187	2.60	2.59	2.00	34,008	3.52	3.52	2.23	<0.0001
	45-64y	1,204,667	3.67	3.67	2.30	285,993	4.36	4.36	2.40	<0.0001
	65-84y	830,660	3.98	3.98	2.38	223,457	4.60	4.60	2.46	<0.0001
Sex										
	Male	1,191,169	3.76	3.54	2.33	273,209	4.46	4.24	2.42	<0.0001
	Female	1,100,123	3.62	3.38	2.34	288,535	4.35	4.14	2.43	<0.0001
Hypertension		1,364,072	4.75	4.53	2.19	392,364	5.21	5.03	2.28	<0.0001
CAD		1,075,682	4.82	4.57	2.28	334,882	5.25	5.07	2.37	<0.0001
Dyslipidemia		1,049,147	4.93	4.70	2.27	279,756	5.55	5.36	2.33	<0.0001
Stroke		451,639	4.62	4.25	2.36	152,623	5.20	4.92	2.47	<0.0001
Osteoporosis		299,885	4.41	4.09	2.33	106,336	4.94	4.69	2.47	<0.0001
DPN		285,704	4.50	4.15	2.44	89,512	5.14	4.86	2.55	<0.0001
DKD		89,931	4.56	4.23	2.57	30,818	5.34	5.05	2.60	<0.0001
DR		98,571	4.85	4.50	2.58	28,456	5.71	5.40	2.56	<0.0001
DA		67,862	4.86	4.47	2.46	21,497	5.53	5.22	2.53	<0.0001
Number of Comorbidities										
	0	484,248	1.51	1.48	1.11	69,066	1.77	1.74	1.11	<0.0001
	1	486,302	2.82	2.76	1.58	104,203	3.07	3.02	1.58	<0.0001
	2	516,990	3.90	3.81	1.89	129,029	4.16	4.09	1.88	<0.0001
	3	533,601	5.19	5.06	2.15	153,233	5.45	5.36	2.19	<0.0001
	4	231,415	5.77	5.64	2.17	87,064	6.13	6.04	2.30	<0.0001
	5	38,736	6.20	6.09	2.20	19,149	6.64	6.55	2.42	<0.0001
Number of Complications										
	0	1,854,980	3.51	3.31	2.27	428,332	4.18	3.99	2.34	<0.0001
	1	344,723	4.27	3.96	2.45	102,302	4.93	4.68	2.51	<0.0001
	2	78,308	5.09	4.70	2.46	25,778	5.76	5.45	2.53	<0.0001
	3	12,395	5.72	5.28	2.43	4,903	6.30	5.95	2.51	<0.0001
	4	886	6.23	5.74	2.44	429	6.82	6.44	2.52	<0.0001

a, multivariable regression models, included covariables: age, sex, hypertension, CAD, dyslipidemia, stroke, osteoporosis, DPN, DKD, DR, DA and year; 0, absence of the disease; 1: presence of the disease; CRD, chronic respiratory disease; CAD, coronary atherosclerotic heart disease; DPN, diabetic peripheral neuropathy; DKD, diabetic kidney disease; DR, diabetic retinopathy; DA, diabetic angiopathies; 0,1,2,3,4,5: the number of comorbidities or complications.

**Table 4:** Total annual drugs cost between non-CRD and CRD in diabetes patients.

				Non-CRD				CRD		*Wilcoxon test*
		*n*	Mean	Adjusted Mean^a^	SD	*n*	Mean	Adjusted Mean^a^	SD	*p* value
Age										
	≥85y	55,778	10,379	10,389	10,131	18,286	12,376	12,372	10,632	<0.0001
	16-44y	200,187	7,403	7,371	9,469	34,008	10,000	9,997	10,033	<0.0001
	45-64y	1,204,667	9,866	9,857	9,730	285,993	12,373	12,377	11,144	<0.0001
	65-84y	830,660	10,558	10,559	9,822	223,457	12,958	12,956	10,873	<0.0001
Sex										
	Male	10,030	9,615	9,923	273,209	12,570	11,976	10,995	10,030	<0.0001
	Female	9,790	9,284	9,637	288,535	12,359	11,758	10,959	9,790	<0.0001
Hypertension		1,364,072	12,221	11,818	9,962	392,364	14,377	13,861	11,360	<0.0001
CAD		1,075,682	12,986	12,553	10,205	334,882	15,081	14,634	11,629	<0.0001
Dyslipidemia		1,049,147	13,289	12,918	10,287	279,756	16,055	15,565	11,743	<0.0001
Stroke		451,639	12,776	12,024	10,648	152,623	15,359	14,597	12,261	<0.0001
Osteoporosis		299,885	12,089	11,417	10,559	106,336	14,468	13,757	11,955	<0.0001
DPN		285,704	12,333	11,632	10,873	89,512	15,004	14,237	12,465	<0.0001
DKD		89,931	13,946	13,211	12,294	30,818	17,606	16,709	13,930	<0.0001
DR		98,571	13,552	12,851	11,343	28,456	17,072	16,191	13,153	<0.0001
DA		67,862	13,450	12,635	11,634	21,497	16,342	15,458	12,597	<0.0001
Number of Comorbidities										
	0	484,248	4,815	4,758	8,114	69,066	5,573	5,481	7,176	<0.0001
	1	486,302	7,279	7,217	7,561	104,203	8,232	8,115	7,727	<0.0001
	2	516,990	10,062	9,990	8,630	129,029	11,212	11,067	9,264	<0.0001
	3	533,601	13,569	13,472	9,985	153,233	15,207	15,015	11,114	<0.0001
	4	231,415	16,009	15,953	11,006	87,064	18,156	17,960	12,407	<0.0001
	5	38,736	18,035	18,048	12,253	19,149	20,901	20,733	14,121	<0.0001
Number of Complications										
	0	1,854,980	9,319	8,932	9,357	428,332	11,564	11,079	10,277	<0.0001
	1	344,723	11,825	11,203	10,807	102,302	14,539	13,854	11,990	<0.0001
	2	78,308	14,425	13,639	11,580	25,778	17,594	16,724	13,827	<0.0001
	3	12,395	16,691	15,791	12,468	4,903	19,831	18,824	13,782	<0.0001
	4	886	19,803	18,715	16,682	429	21,342	20,307	16,277	<0.0001

a, multivariable regression models, included covariables: age, sex, hypertension, CAD, dyslipidemia, stroke, osteoporosis, DPN, DKD, DR, DA and year; 0, absence of the disease; 1: presence of the disease; CRD, chronic respiratory disease; CAD, coronary atherosclerotic heart disease; DPN, diabetic peripheral neuropathy; DKD, diabetic kidney disease; DR, diabetic retinopathy; DA, diabetic angiopathies; 0,1,2,3,4,5: the number of comorbidities or complications.

### The difference in diabetes therapy regimens between CRD and non-CRD patients

In terms of insulin, diabetes patients used premixed insulin more often with a proportion of 53.8% in the nonrespiratory series and 59.9% in the respiratory group in 2018. Diabetes patients with CRD used less fast-acting (10.0% vs. 12.4%, 2018, *p* <.0001), less long-acting (25.2% vs. 29.8%, 2018, *p* <.0001), more premixed (59.9% vs. 53.8%, 2018, *p* <.0001), and more intermediated-acting insulin (12.7% vs. 12.3%, 2018, *p* = .0061). There was no difference in the rate of short-acting insulin use between them. The trends in 2016 and 2017 were similar to those in 2018 (**Table 5**).

**Table 5:** Different type of insulin used between non-CRD and respiratory from 2016 to 2018.

Type of Insulin			Non-CRD			CRD		2016	2017	2018
		2016 (%)	2017 (%)	2018 (%)	2016 (%)	2017 (%)	2018 (%)	*p* value	*p* value	*p* value
Fast-acting								<0.0001	<0.0001	<0.0001
	0	174515 (91.4%)	176352 (89.3%)	177418 (87.6%)	47921 (93.3%)	53162 (91.6%)	48501 (90.0%)			
	1	16477 (8.6%)	21156 (10.7%)	25094 (12.4%)	3429 (6.7%)	4893 (8.4%)	5403 (10.0%)			
Short-acting								0.8202	0.3799	0.6947
	0	166490 (87.2%)	174554 (88.4%)	180930 (89.3%)	44743 (87.1%)	51385 (88.5%)	48191 (89.4%)			
	1	24502 (12.8)	22954 (11.6%)	21582 (10.7%)	6607 (12.9%)	6670 (11.5%)	5713 (10.6%)			
Intermediate-acting								0.0013	0.0177	0.0061
	0	161971 (84.8%)	170623 (86.4%)	177645 (87.7%)	43251 (84.2%)	49929 (86%)	47049 (87.3%)			
	1	29021 (15.2%)	26885 (13.6%)	24867 (12.3%)	8099 (15.8%)	8126 (14%)	6855 (12.7%)			
Long-acting								<0.0001	<0.0001	<0.0001
	0	149763 (78.4%)	147120 (74.5%)	142126 (70.2%)	42066 (81.9%)	45673 (78.7%)	40335 (74.8%)			
	1	41229 (21.6%)	50388 (25.5%)	60386 (29.8%)	9284 (18.1%)	12382 (21.3%)	13569 (25.2%)			
Premixed								<0.0001	<0.0001	<0.0001
	0	77379 (40.5%)	84465 (42.8%)	93467 (46.2%)	18327 (35.7%)	21512 (37.1%)	21608 (40.1%)			
	1	113613 (59.5%)	113043 (57.2%)	109045 (53.8%)	33023 (64.3%)	36543 (62.9%)	32296 (59.9%)			

Diabetes patients with CRD used more antidiabetic drugs than the nonrespiratory disease group (93.1% *vs* 88.1%, 2018, *p* <.0001). The CRD patients used more oral combination therapy (55.3% vs 50.8%, 2018, *p* <.0001) and combinations of oral drugs and insulin (24.6% *vs* 20.7%, 2018, *p* <.0001); the proportion of monotherapy was lower than in the non-CRD group (32.6% *vs* 37.5%, 2018, *p* <.0001) (**Table 6**). The most commonly used drugs were AGIs and metformin, whether as a monotherapy or combination.

**Table 6:** Difference of therapy regimens between non-CRD and CRD from 2016 to 2018.

			Non-CRD			CRD		2016	2017	2018
		2016 (%)	2017 (%)	2018 (%)	2016 (%)	2017 (%)	2018 (%)	*p* value	*p* value	*p* value
Receiving any antidiabetic drugs										
	0	106,739 (14.8)	102,620 (13.5)	96,476 (11.9)	16,148 (9.2)	16,032 (8.1)	13,075 (6.9)	<0.0001	<0.0001	<0.0001
	1	614,661 (85.2)	659,901 (86.5)	710,895 (88.1)	159,837 (90.8)	180,956 (91.9)	175,696 (93.1)	<0.0001	<0.0001	<0.0001
Monotherapy		255,070 (41.5)	257,735 (39.1)	266,345 (37.5)	56,810 (35.5)	61,069 (33.7)	57,306 (32.6)	<0.0001	<0.0001	<0.0001
	AGIs	90,304 (14.7)	89,454 (13.6)	89,304 (12.6)	20,882 (13.1)	22,009 (12.2)	19,960 (11.4)	<0.0001	<0.0001	<0.0001
	Metformin	77,115 (12.5)	87,294 (13.2)	98,730 (13.9)	17,051 (10.7)	20,599 (11.4)	21,178 (12.1)	<0.0001	<0.0001	<0.0001
	SUs	24,476 (4.0)	23,020 (3.5)	21,314 (3.0)	5,943 (3.7)	5,894 (3.3)	5,131 (2.9)	0.7417	0.534	0.0571
	Premixed insulin	37,467 (6.1)	31,910 (4.8)	28,107 (4.0)	8,136 (5.1)	7,588 (4.2)	6,031 (3.4)	<0.0001	<0.0001	<0.0001
	DPP-4i	0 (0.0)	2,255 (0.3)	6,028 (0.8)	0 (0.0)	244 (0.1)	834 (0.5)	-	<0.0001	<0.0001
	Glinides	8,195 (1.3)	7,091 (1.1)	6,306 (0.9)	1,787 (1.1)	1,638 (0.9)	1,320 (0.8)	<0.0001	<0.0001	0.0002
Oral combination therapy		275,643 (44.8)	317,618 (48.1)	360,982 (50.8)	81,166 (50.8)	96,290 (53.2)	97,143 (55.3)	<0.0001	<0.0001	<0.0001
	AGIs + Metformin	54,613 (8.9)	65,404 (9.9)	75,018 (10.6)	15,282 (9.6)	18,978 (10.5)	19,459 (11.1)	<0.0001	<0.0001	<0.0001
	AGIs + SUs	41,083 (6.7)	39,072 (5.9)	36,648 (5.2)	11,363 (7.1)	11,346 (6.3)	9,566 (5.4)	<.0001	<0.0001	<0.0001
	Metformin + SUs	36,435 (5.9)	39,393 (6.0)	40,454 (5.7)	10,016 (6.3)	11,007 (6.1)	10,580 (6.0)	<0.0001	<0.0001	<0.0001
	AGIs + Metformin + SUs	34,275 (5.6)	39,036 (5.9)	41,721 (5.9)	11,238 (7.0)	12,990 (7.2)	12,486 (7.1)	<0.0001	<0.0001	<0.0001
	Metformin + DPP-4i	0 (0.0)	4,006 (0.6)	11,066 (1.6)	0 (0.0)	559 (0.3)	1,691 (1.0)	-	<0.0001	<0.0001
	Metformin + Glinides	9,818 (1.6)	9,486 (1.4)	9,006 (1.3)	2,434 (1.5)	2,442 (1.3)	2,071 (1.2)	0.4736	0.8762	0.4931
	AGIs + Glinides	8,293 (1.3)	7,667 (1.2)	6,948 (1.0)	2,148 (1.3)	2,088 (1.2)	1,665 (0.9)	0.0128	0.0316	0.3648
Oral + Insulin		126,163 (20.5)	138,755 (21.0)	147,085 (20.7)	38,249 (23.9)	45,440 (25.1)	43,209 (24.6)	<0.0001	<0.0001	<0.0001
	AGIs + Premixed insulin	26,692 (4.3)	25,615 (3.9)	23,266 (3.3)	7,355 (4.6)	7,862 (4.3)	6,634 (3.8)	<0.0001	<0.0001	<0.0001
	AGIs + Metformin + insulin	13,467 (2.2)	16,613 (2.5)	17,563 (2.5)	4,557 (2.9)	6,298 (3.5)	5,833 (3.3)	<.0001	<0.0001	<0.0001
	Metformin +Premixed insulin	14,256 (2.3)	14,985 (2.3)	14,708 (2.1)	4,151 (2.6)	4,570 (2.5)	4,145 (2.4)	<.0001	<0.0001	<0.0001

CRD, chronic respiratory disease; AGIs, alpha-glucosidase inhibitors; SUs, sulfonylureas; DDP-4i, dipeptidyl peptidase-4 inhibitor.

### Medications and medical costs in patients with stratified CRD

When we stratified the CRD into parenchymal lung lesions (including lung cancer and TB) and airway disease (including COPD, tracheitis/bronchitis, and asthma), we found that both parenchymal lung lesions and airway disease had higher insulin use ratios (40.20%, 28.52%, all *p’*s <.0001, respectively). Airway disease had higher medical costs and needed more complicated therapy than monotherapy (**Table 7**).

**Table 7:** Medications and medical costs in Stratified Chronic respiratory disease patients.

	Total CRD	PLL	Airway disease	Non-CRD	*p* 1	*p* 2

*n*	188,771	811	188,165	807,371		
Insulin use rate (%)	53,904 (28.56)	326 (40.20)	53,659 (28.52)	202,512 (25.08)	<0.0001	<0.0001
Number of medications	4.48 ± 2.41	3.30 ± 2.52	4.48 ± 2.41	3.76 ± 2.33	<0.0001	<0.0001
Hypoglycemic drugs	1.93 ± 1.08	1.72 ± 1.20	1.93 ± 1.08	1.71 ± 1.08	0.8282	<0.0001
Nonhypoglycemic drugs	2.55 ± 1.93	1.58 ± 1.94	2.55 ± 1.93	2.05 ± 1.85	<0.0001	<0.0001
Number of comorbidities	2.52 ± 1.53	1.71 ± 1.66	2.52 ± 1.53	2.07 ± 1.50	<0.0001	<0.0001
Glycemic diseases	0.30 ± 0.59	0.26 ± 0.58	0.30 ± 0.59	0.23 ± 0.53	0.1714	<0.0001
Nonglycemic diseases	2.22 ± 1.32	1.45 ± 1.43	2.23 ± 1.32	1.84 ± 1.33	<0.0001	<0.0001
Total annual drug cost, ¥	12,286 ± 10,385	10,362 ± 15,884	12,297 ± 10,383	9,700 ± 9,202	0.0460	<0.0001
Hypoglycemic drugs	5,906 ± 7,111	6,515 ± 14,370	5,907 ± 7,106	5,188 ± 6,985	<0.0001	<0.0001
Nonhypoglycemic drugs	6,380 ± 6,670	3,847 ± 5,806	6,391 ± 6,672	4,512 ± 5,288	0.0007	<0.0001
Total annual cost/drug, ¥	2,706 ± 2,106	2,857 ± 3,906	2,706 ± 2,102	2,493 ± 2,518	<0.0001	<0.0001
Cost/hypoglycemic drug/)	2,800 ± 3,058	2,988 ± 5,802	2,800 ± 3,046	2,624 ± 3,228	0.0012	<0.0001
Cost/nonhypoglycemic drug	2,148 ± 2,104	1,461 ± 2,474	2,151 ± 2,102	1,661 ± 1,837	0.0027	<0.0001
Antidiabetic therapy regimen						
Receiving any antidiabetic drugs (%)	175,696(93.07)	681(83.97)	175,198(93.11)	710,895(88.05)	0.0004	<0.0001
Monotherapy (%)	57,306(32.62)	231(33.92)	57,129(32.61)	266,345(37.47)	0.0563	<0.0001
Oral combination therapy (%)	97,143(55.29)	300(44.05)	96,932(55.33)	360,982(50.78)	0.0005	<0.0001
Oral + Insulin (%)	43,209(24.59)	202(29.66)	43,068(24.58)	147,085(20.69)	<0.0001	<0.0001

CRD, chronic respiratory disease; PLL, parenchymal lung lesions, including lung cancer and tuberculosis; airway disease, including COPD, tracheitis/bronchitis, and asthma; p 1, PLL vs. non-CRD; p 2, airway disease vs. non-CRD.

### Changes in antidiabetic drugs and costs

**Figure 2:**
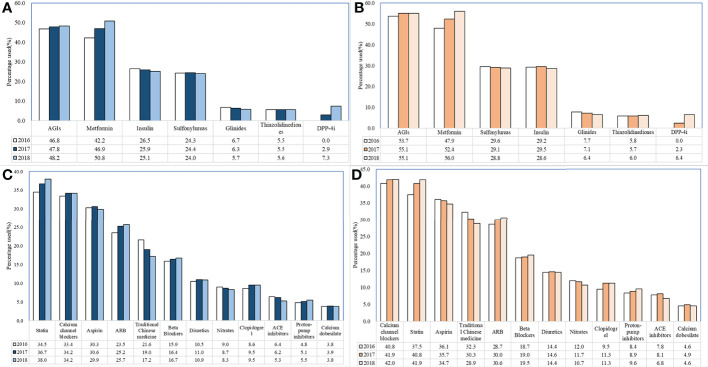
Changes in use of nonhypoglycemic drugs and hypoglycemic drugs in DM patients with or without chronic respiratory disease. **(A)**. Changes in use of hypoglycemic drugs (nonchronic respiratory diseases) **(B)**. Changes in use of hypoglycemic drugs (chronic respiratory diseases) **(C)**. Changes in use of nonhypoglycemic drugs (nonchronic respiratory diseases) **(D)**. Changes in use of nonhypoglycemic drugs (chronic respiratory diseases).

Whether diabetes patients combined with CRD or not, AGIs and metformin were the most frequently prescribed hypoglycemic drug classes: 46.8% (2016), 47.8% (2017), and 48.2% (2018) for AGIs; 42.2% (2016), 46.9% (2017), and 50.8% (2018) for metformin; and in the nonrespiratory series, 53.7% (2016), 55.1% (2017), and 55.1% (2018) for AGIs; 47.9% (2016), 52.4% (2017), and 56% (2018) for metformin. Among the nonhypoglycemic drugs, the most significant change was observed in the use of traditional Chinese medicines (**Figure 2**).

From 2016 to 2018, diabetes patients with CRD used more medications (4.30 in 2016 and 4.48 in 2018, a 4.2% increase) and suffered fewer comorbidities (2.57 in 2016 and 2.52 in 2018, a 1.9% decrease), whereas total annual drug costs were reduced (¥12,989 in 2016, ¥12,160 in 2017, and ¥12,286 in 2018, a 5.4% reduction) (**Table 2**). When we analyzed the diabetes patients with CRD, it was found that the use of fast- and long-acting insulin increased gradually, whereas premixed and shorting- and intermediate-acting insulin use decreased significantly (*p* <.0001 for 2016 vs. 2017, 2017 vs. 2018; **Table 5**).

## Discussion

In this observational study, we compared the characteristics, treatment costs, and medications of diabetes patients with and without CRD. We found that diabetes patients with CRD used more medications and had higher total annual drug costs to treat more comorbidities than those without CRD, respectively. We also showed the changes in medication increases and cost reductions in diabetes patients with CRD from 2016 to 2018. To date, this study represents Asia’s largest sample of studies on diabetes medications and medical costs combined with CRD.

Numerous studies on the cost of treatment for diabetes patients have been conducted worldwide. Li et al. enrolled 871 type 2 diabetes patients and showed that the annual mean DMC was $1990.20 in China (1). Prior studies report that the annual mean indirect costs were nearly $206.10 (23). Diabetes imposes a significant financial burden due to its high prevalence (6), low awareness of diagnosis, and inadequate glycemic control (10), and diabetes imposes a significant financial burden with medical expenditures approximately 2.3 times higher than those without diabetes (5). Our study examined diabetes patients with Beijing Medical Insurance to identify whether or not CRD had an effect. Remarkably, these data were collected between 2016 and 2018, excluding the heterogeneity caused by COVID-19, which resulted in increased diabetes medical costs clearly (24).

A previous study based on the Biobank Japan Project revealed that 4.45% (1373/30,834) of type 2 diabetes patients have a combination of COPD, interstitial pneumonia, pulmonary fibrosis, and pneumoconiosis (14). Our finding showed that 18.95%–20.53% of diabetes patients suffered CRD, which is higher than the overall population prevalence (25). In general, diabetes patients have a significant association with the risk of death from CRD, particularly from respiratory infection (17). Similar studies (18, 26–29) conducted in different areas also confirmed the link between diabetes and higher risk prevalence and mortality from CRD such as TB, COPD, and obstructive sleep apnea, among others. Diabetes is also an unfavorable prognostic factor for COPD (30). Our study identified the types of CRD as COPD, asthma, tracheitis/bronchitis, TB, and lung cancer and observed the higher prevalence of comorbidities or complications, such as hypertension, CHD, dyslipidemia, stroke, DPN, DKD, DR, and DA, among others (**Table 1**). As expected, the medications and costs for treating more comorbidities and complications were higher than in diabetes without CRD.

Although men outnumbered women in the diabetes population, we uncovered women as a risk factor for CRD among patients with diabetes, consistent with previous research findings (14, 17, 22). Men were more affected by severe community-acquired pneumonia (31) due to a higher proportion of smokers (32). Women have higher compliance than men, which may also affect the ratio. The gender difference in diabetes with CRD needs more research including different populations.

Previous research studied 9872 type 2 diabetes outpatients and found that metformin (53.7%) was the most commonly used OAD in China, followed by SUs (42.7%) and AGIs (35.9%) (9). Obviously, as the primary treatment, metformin was used in a widespread manner in different areas (9, 33, 34). When we compared the therapy regimens of non-CRD and CRD patients, AGIs and metformin remained the most useful OADs, whether patients suffered from CRD (11.4%, 12.1%, respectively) or not (12.6%, 13.9%, respectively) in 2018. This phenomenon could be attributed primarily to dietary structure and blood glucose levels. In China, diabetes patients showed a higher prevalence of isolated impaired glucose tolerance than isolated impaired fasting glucose (35), and AGIs verified a better efficacy for the postprandial blood glucose levels. In addition, 32.6% of diabetes patients with CRD received monotherapy glycemic treatment to achieve a satisfactory level, whereas the proportion could reach 37.5% in diabetes patients without CRD. Alwhaibi et al. found that polypharmacy in diabetes patients (who received more than five drugs) was 2.41 times higher in diabetes patients with CRD than without CRD (36). Despite some sterile inflammation, diabetes patients with CRD use antibiotics and inhaled glucocorticoids as needed, increasing the prevalence of insulin resistance (37) and leading to an increase in antidiabetic treatment, especially in airway disease.

Our data showed that diabetes patients with CRD received premixed insulin more often (59.9% vs. 53.8%, *p* <.0001) and received less fast- (10.0% vs. 12.4%, *p* <.0001) and long-acting insulin (25.2% vs. 29.8%, *p* <.0001) although premixed insulin had a higher risk of hypoglycemia than basal-and-bolus insulin (BBI) (38). Physicians prefer premixed insulin as a short-term simplified intensification therapy, and its use increases when patients have poor glycemic control, including diabetes complicated with CRD. Furthermore, unlike in Western countries, the Chinese were accustomed to consuming more carbohydrates, resulting in higher postprandial blood glucose levels. The convenience and effectiveness of premixed insulin contribute significantly to its increased use (39). The study conducted a subgroup analysis, which revealed that diabetes patients with COPD had a more pronounced survival advantage for insulin adherence (insulin use rate ≥90%) (40). This may be a reminder of the importance of standardized insulin therapy, particularly in diabetes patients with CRD in the future.

As the new patterns of hypoglycemic drugs manifested, such as sodium-glucose cotransporter 2 inhibitor (SGLT2i), glucagon-like peptide-1 receptor agonist (GLP-1RA), and dipeptidyl peptidase-4 inhibitors (DDP4i), the use of the antidiabetic medication class has shifted (33). Albogami et al. performed real-world data spanning and uncovered that potentially beneficial effects of GLP-1RA should be considered in the selection of the hypoglycemic treatment regimen (41). In China, DDP4i, SGLT2i, and GLP-1RA were admitted to the Beijing Medical Insurance catalogue in 2017, 2020, and 2020, respectively, and we did not analyze the DDP4i and GLP-1RA in this research.

DMC typically increases as the number of the complications increases (23, 42) as does the number of outpatient visits (42). Yang et al. used 10 years of a longitudinal observational study of diabetes patients to discover that the three most expensive complications were end-stage renal disease ($97,431 for type 1 diabetes, $98,981 for type 2 diabetes), congestive heart failure ($14,855 for type 1 diabetes, $7062 for type 2 diabetes), and myocardial infarction ($9496 for type 1 diabetes, $8572 for type 2 diabetes) (12). Another study conducted in Beijing, China, found the similar trend in hospitalization costs as well as a rise in the number of complications and length of hospital stay; however, the diabetic foot was the most expensive hospitalization (8). According to our data, whether diabetes was comorbid with CRD or not, medications and costs have risen as the number of complications has increased.

From 2016 to 2018, the medications increased and the medical cost decreased in diabetes patients with CRD. This is attributed to actualizing the standard guidelines and the full implementation of the Zero-Markup Medicine Policy (ZMDP) in 2016 in Beijing, China, which was demonstrated in decreasing the medical cost of drugs in many chronic, noncommunicable diseases, including type 2 diabetes (43).

The limitations of this study are listed below. The proportion of type 1/type 2 diabetes should be different in patients of different ages layered theoretically, corresponding to different treatment strategies. However, in this retrospective study, we did not perform this stratification to type 1 and type 2 diabetes. Our study only included outpatients with Beijing medical insurance, and we did not analyze patients with confirmed diabetes but who were not treated with prescribed antidiabetic medications. The economy in Beijing is more developed than most areas in China, so our statistics may not be representative of the current situation in China. In addition, we cannot obtain the laboratory indices to evaluate the prognoses. Along with the emergence of new antidiabetic medications, which have proven proper heart and kidney benefits, a cost-effectiveness analysis with larger sample sizes will be needed in the future all over the world.

In conclusion, the retrospective study analyzed the hypoglycemic treatment regimen and costs with a large sample size based on Beijing Medical Insurance. Diabetes patients with CRD series suffered from more comorbidities and were treated with more hypoglycemic drugs and medical costs. The comorbidities and numbers of medications increased and the total medical costs increased when combined with CRD. Diabetes patients with CRD showed an increase in medications and reduction in costs from 2016 to 2018. This may be a reminder of the importance of antidiabetic therapy, particularly in diabetes patients with chronic respiratory disease, in the future.

## Data availability statement

The raw data supporting the conclusions of this article will be made available by the authors, without undue reservation.

## Ethics statement

The studies involving human participants were reviewed and approved by Beijing Hospital. The patients/participants provided their written informed consent to participate in this study.

## Author contributions

LG, JQ, ZT, and XX designed the study and wrote the first draft of the manuscript. WW and JL analyzed and interpreted the data. QP, JQ, YZ, and JF devised the whole project and critically reviewed the manuscript. QP and JQ revised the manuscript. All authors read and approved the final manuscript.

## Funding

This study was supported by the National key research and development program (2020YFC2009000).

## Conflict of interest

The authors declare that the research was conducted in the absence of any commercial or financial relationships that could be construed as a potential conflict of interest.

## Publisher’s note

All claims expressed in this article are solely those of the authors and do not necessarily represent those of their affiliated organizations, or those of the publisher, the editors and the reviewers. Any product that may be evaluated in this article, or claim that may be made by its manufacturer, is not guaranteed or endorsed by the publisher.

## Abbreviations

CRD, chronic respiratory disease; DKD, diabetic kidney disease; DR, diabetic retinopathy; DPN, diabetic peripheral neuropathy; BMI, body mass index; CAD, coronary atherosclerotic heart disease; SAHS, sleep apnea-hypopnea syndrome; DA, diabetic angiopathies; OADs, oral antidiabetic drugs; AGIs, α-glucosidase inhibitors; SUs, sulfonylurea.
